# On Your Marks, Get Set, Recover: Psychological Sequelae of Sports Injury and Evidence-Based Interventions for Return to Sport

**DOI:** 10.1192/bjo.2026.11196

**Published:** 2026-06-30

**Authors:** Nour Al Tarsha

**Affiliations:** University of Liverpool, Liverpool, United Kingdom

## Abstract

**Aims:**

Psychological factors play a critical role in rehabilitation after sports injury and may delay return to sport (RTS) despite physical recovery. However, the most prevalent psychological sequelae and the comparative effectiveness of rehabilitation strategies are not consistently integrated into clinical pathways. This study aims to identify common psychological outcomes following sports trauma and determine which evidence-based psychological interventions are most strongly associated with improved rehabilitation and RTS outcomes. We hypothesised that (1) functional psychological symptoms would be more prevalent than formal psychiatric diagnoses and (2) cognitive-behavioural approaches would show the strongest association with positive recovery outcomes.

**Methods:**

A structured review of peer-reviewed literature published between 2014 and 2025 was conducted. PubMed and Google Scholar were searched using predefined keywords related to sports injury, psychological outcomes, and rehabilitation interventions. Studies were included if they assessed psychological symptoms, return-to-sport outcomes, or intervention effectiveness in injured athletes. Findings were synthesised narratively, with intervention effects grouped by therapeutic modality.

**Results:**

Functional psychological sequelae were common following sports injury, particularly fear of reinjury (50%), reduced confidence (45%), and sleep disturbance or hyperarousal (40%). These were more frequent than formally diagnosed psychiatric disorders such as PTSD (25%) or major depressive disorder (35%). Anxiety symptoms were the strongest psychological predictor of delayed RTS, while reduced confidence was consistently associated with poorer rehabilitation outcomes. Depressive symptoms were most evident during prolonged inactivity and career-ending injuries, where loss of athletic identity (25%) was prominent.

Among psychological interventions, cognitive behavioural therapy (CBT) demonstrated the highest reported effectiveness in reducing psychological symptoms and supporting RTS (28.6%, 95% CI [20.6–36.6]). Graded exposure showed the next greatest effect (23.2%, 95% CI [13.2–33.2]), followed by psychological skills training (17.9%, 95% CI [8.9–26.9]). Stress-management interventions improved adherence to physical rehabilitation, whereas arousal imagery techniques were linked to increased pre-RTS anxiety in some athletes.

**Conclusion:**

Functional psychological symptoms are more prevalent than formal psychiatric diagnoses after sports injury, supporting our first hypothesis, while cognitive-behavioural interventions show the strongest association with improved psychological outcomes and RTS readiness, supporting our second. Routine psychological screening and targeted CBT-based support may enhance rehabilitation. A biopsychosocial approach incorporating ongoing psychological assessment, pre-injury profiling tools (e.g. Sport Mental Health Assessment Tool-1; Athletic Coping Skills Inventory-28) and a 4Ps framework supports tailored, preventative rehabilitation and promotes resilient RTS. Screening and data extraction by a single reviewer increases risk of selection bias.

